# Bioactive Components in Fish Venoms

**DOI:** 10.3390/toxins7051497

**Published:** 2015-04-30

**Authors:** Rebekah Ziegman, Paul Alewood

**Affiliations:** Institute for Molecular Bioscience, the University of Queensland, St. Lucia, QLD 4072, Australia

**Keywords:** fish venom, venom proteins, venom peptides, pharmacology, pore forming toxins, stonefish toxins

## Abstract

Animal venoms are widely recognized excellent resources for the discovery of novel drug leads and physiological tools. Most are comprised of a large number of components, of which the enzymes, small peptides, and proteins are studied for their important bioactivities. However, in spite of there being over 2000 venomous fish species, piscine venoms have been relatively underrepresented in the literature thus far. Most studies have explored whole or partially fractioned venom, revealing broad pharmacology, which includes cardiovascular, neuromuscular, cytotoxic, inflammatory, and nociceptive activities. Several large proteinaceous toxins, such as stonustoxin, verrucotoxin, and Sp-CTx, have been isolated from scorpaenoid fish. These form pores in cell membranes, resulting in cell death and creating a cascade of reactions that result in many, but not all, of the physiological symptoms observed from envenomation. Additionally, Natterins, a novel family of toxins possessing kininogenase activity have been found in toadfish venom. A variety of smaller protein toxins, as well as a small number of peptides, enzymes, and non-proteinaceous molecules have also been isolated from a range of fish venoms, but most remain poorly characterized. Many other bioactive fish venom components remain to be discovered and investigated. These represent an untapped treasure of potentially useful molecules.

## 1. Introduction

Animal venoms have long been considered an excellent resource for the discovery of novel, biologically active molecules. There has been much research on the activities and components of terrestrial venoms, such as those from snakes, scorpions, and spiders but relatively less into marine and aquatic venoms [1,2,3,4,5,6]. This is due in part to the greater convenience in capturing terrestrial animals over marine specimens, and partly because, as terrestrial animals ourselves, marine organisms are viewed as less of a threat [5]. However, many venomous animals can be found in marine environments and some are capable of producing severe envenomation in humans that have led to fatalities [7,8,9,10,11,12]. Many of these species are invertebrates from the cnidarian and conus families. However, fish venom research is underrepresented in the literature, possibly because of the extreme lability of some venom components [13]. Additionally, in some fishes, mucus can contaminate venom samples, presenting a major challenge to their study [14,15]. Nonetheless, fish venoms represent a largely untapped treasure of biologically important compounds.

**Figure 1:**
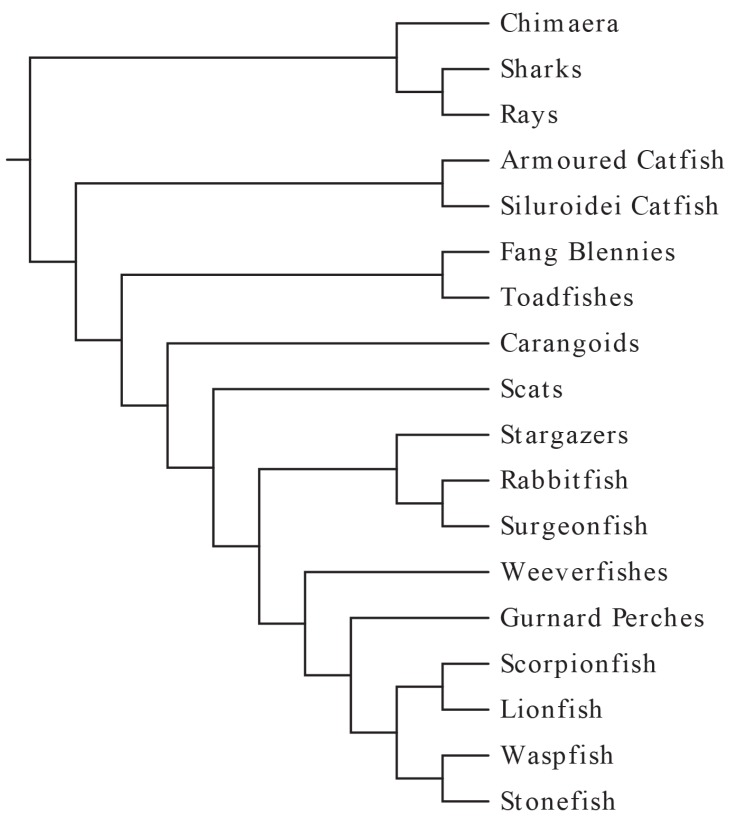
A phylogenetic tree showing the known clades of venomous fishes. Adapted from Smith and Wheeler [16] and Wright [17] by author and assembled using EvolView [18].

Although the number of venomous fish species was once thought to be around 200 [5], a more recent study examining phylogeny and venom evolution in ray-finned fishes has raised that estimate to as many as 2000 [16], which may itself be low considering a recent study suggesting that there may be over 1600 venomous catfish species alone [17]; inclusion of cartilaginous fishes would raise this estimate even higher. Altogether, fish comprise more than half of all venomous vertebrates [16]. Interestingly, the venom apparatus and pharmacology are similar throughout most venomous fish species, despite their wide taxonomic range (Figure 1).

**Figure 2:**
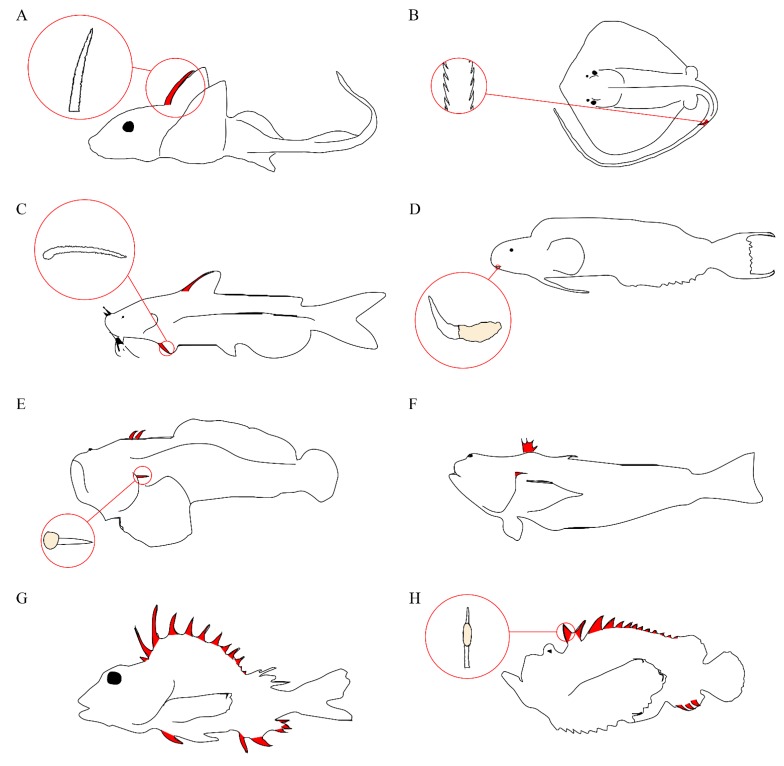
Representative venomous fishes. Venomous spines on fish are colored in red. (**A**) Chimaera and serrated dorsal spine; (**B**) Stingray and serrated caudal spine; (**C**) Catfish and serrated pectoral spine; (**D**) Fang blenny and canine tooth with venom gland; (**E**) Toadfish and opercular spine with venom gland; (**F**) Weeverfish; (**G**) Gurnard Perch; (**H**) Stonefish and dorsal spine with venom gland.

Venom apparatus have arisen 11 separate times in modern acanthomorphs and twice in catfish [17], with venomous species representing at least four different teleost orders [16]. The venom apparatus of fishes typically consists of venom glands located in paired anterolateral grooves on either side of sharp spines, with the spine and venom gland complex covered by an integumentary sheath. The venomous spines are most often found in association with the dorsal fin, but pelvic, anal, and pectoral spines are also common (Figure 2). Some species, notably stargazers, toadfish, and weeverfish, possess venomous opercular spines on either side of their heads. In addition to opercular spines, toadfish and weeverfish also maintain venomous dorsal spines. In venomous toadfish, the glandular venom tissue sits at the base of these spines. Stingrays lack all of these and instead produce serrated spines in their caudal region.

Fish venom glands can vary greatly in respect to size and cellular morphology, even between closely related species [17]. However, most of them do appear to maintain certain similarities. Typically, the glands are composed of large glandular cells surrounded by supporting cells, which provide stability to the gland and connect it to the surrounding tissue [19]. Evidence points to a holocrine secretion method for the venom [20,21,22]. Additionally, histology often shows the presence of cytoplasmic granules on the internal surface of the gland cell membranes [23], and smaller granules could also be found in the vacuoles contained in the gland cells [24]. In stingrays, epidermal tissue covers the serrated stings, and both secretory cells specialized for the sting and secretory cells that can be found throughout the rest of the animals’ epidermis are present [25], which is similar to the glandular epithelium that was found to cover the venom spine of the pacific ratfish *Hydrolagus colliei* [26].

Toadfishes of the subfamily Thalassophryninae possess the most highly developed venom apparatus of the fishes, in which the venomous dorsal and opercular spines manifest themselves as enclosed hollow tubes leading out from the venom glands [27]. Fang blenny species are unique in that their venom apparatus utilizes venomous canine teeth, though the buccal glandular tissue resembles the dermal glandular tissue of other venomous species [22]. In stonefish, the venom gland is more advanced, with the distal end attenuated to form a duct-like structure within the spine groove through which the venom may travel before being expelled from the spine [24]. Envenomation occurs when mechanical pressure is applied to the venom apparatus, causing the venom to be expelled through the canaliculated spines and into the victim [28]. Venomous fish lack musculature associated with their venom apparatus, and are therefore unable to voluntarily control the release of their venom.

As fish spines are modified scales, if a spine were to break off during an envenomation, the fish is able to regenerate the spine and associated venom gland [29]. This is useful to researchers looking to study the venom, as fish may be kept alive and milked periodically. Venom can be obtained by clipping off the entire spine and venom gland apparatus and homogenizing it, and then extracting the venom via centrifugation [30]. However, this ensures that it will take a longer period of time for the fish to regenerate the venom apparatus, and may result in the degradation of more labile venom components. More efficient methods include inserting a syringe directly into the venom gland to remove venom [29,31], or pushing membrane-covered Eppendorf tubes over the venomous spines, forcing the venom into the tube. However, for fish venoms containing a greater concentration of contaminating mucus, additional precipitation methods may be required [14].

Because the venom apparatus of fish are relatively primitive, they are thought to have been acquired fairly recently in evolutionary history and serve purely defensive purposes consistent with their involuntary expulsion mechanism. The development of such a highly effective defense mechanism has allowed many venomous fish to adapt a sedentary lifestyle wherein they camouflage themselves among the rocks and detritus on the sea floor, erecting their venomous spines when perceived threats are near. Possible exceptions to this are the rare, deep-sea Monognathid eels. This group has evolved a singular venomous fang at the front of the skull, which is thought to serve in disabling prey [32,33] though information is sparse.

As the vast majority of fish venoms appear to have evolved as a defensive strategy against other vertebrate species, it stands to reason that envenomation events in humans can have severe effects. Indeed, envenomation incurs a large range of symptoms that have occasionally been known to cause fatalities [7,34]. The most notable symptom is extreme pain disproportionate to the size of the injury [5]. The pain, in addition to being severe, may also radiate up the affected limb to the regional lymphatics [35,36,37,38]. Edema and erythema are also relatively common [39] and in some cases vesicles may form around the wound [40,41]. Systemic symptoms resulting from fish stings include ischemia, muscle spasms, tissue necrosis, prolonged weakness, and nausea, as well as paralysis of the affected limb, hallucinations, loss of perception, hypotension, tachycardia, and respiratory distress. Slow healing and necrosis have been observed following envenomation [42]. It is generally thought that if death is going to occur it will do so within the first several hours following contact [20]. The extent of the damage from envenomation can vary according to the relevant species, number and depth of envenomation sites, and individual reaction to the venom components. Secondary infections are also known to occur, leading to addition damage [36,39,43,44,45].

Although comprehensive data on fish envenomation frequencies is unavailable, case studies make it clear that it is not an uncommon problem, especially among fishermen [12,35,36,37,39,43,45,46]. The treatment for fish envenomation is primarily targeted at relieving the intense pain and involves soaking the affected area in hot water (typically 45–50 °C) for an extended period of time or until the pain subsides. This is thought to denature the noxious proteinaceous components in the venom, though this theory has been challenged based on the observation that in some cases, hot water alleviates pain only while the affected area is immersed [39,47]. In cases where hot water immersion is insufficient, local anesthetics may also be prescribed although morphine has been found to be ineffective in certain clinical cases [34,48] suggesting that the nociceptive activity largely occurs via a non-opiate mechanism. However, Lopes-Ferreira *et al.* [49] demonstrated that, while pretreatment of mice with indomethacin, dexamethasone, cyproheptadine, or L-NAME effected neither the nociceptive nor edematogenic responses to *Thalassophryne nattereri* venom, the opioid analgesic fentanyl did significantly decrease nociception, as demonstrated by paw licking time. Interestingly, the study found that, while fentanyl failed to attenuate the inflammatory response to *T. nattereri* venom, a specific tissue kallikrein inhibitor was able to attenuate both nociception and inflammation by 78% and 24% respectively, whereas a specific plasma kallikrein inhibitor attenuated paw edema by 15% but had no effect on nociception.

Additionally, there is a commercially available horse-derived antivenom against the venom of the stonefish *Synanceia horrida* that can be employed in extreme cases (it is worth noting here that alternative names for this species such as *Synanceja horrida* and *Synanceia trachynis* have been used throughout the literature). Stonefish antivenom (SFAV) has shown cross-reactivity with several other scorpaeniform venoms [50,51], though experiments have shown that it does not cross-react with the venom of the scorpaeniform Bullrout, *Notesthes robusta* [52]. Therefore, it is possible that SFAV could be used to treat some, but not all, envenomations from other scorpaeniform species. An experimental antivenom against *T. nattereri* venom that neutralizes lethality, necrosis, and edema in rabbit and mice has also been developed [53,54]. Since so little is known about the mechanisms by which piscine venoms work, it is nearly impossible to formulate highly effective treatments against them. A better knowledge of the venom components and their mechanisms of action would assist in the development of more effective treatment protocols for envenomation events.

Interestingly, the skin secretions of fish also contain other bioactive components; deemed “ichthyocrinotoxins”, these secretions are presumed to have anti-parasitic activity [55] and may be partially responsible for allowing reduction of squamation in some fish species. While the lack of a specialized delivery system makes it impossible to truly classify ichthyocrinotoxins as venom in their own right [5], they represent an additional resource for new physiologically relevant compounds. In fact, piscine venoms appear to have evolved from ichthyocrinotoxins [56] and antibacterial activity has been found in some fish spine venoms [57,58]. It is likely that during an envenomation event, ichthyocrinotoxin also makes its way into the puncture wound, and may be responsible for some of the resulting symptoms. Nevertheless, piscine venoms and ichthyocrinotoxins contain distinctly different components [58], based on HPLC profiles and subsequent masses from 1D gels and MALDI-ToF analysis. As they do not truly represent venoms, ichthyocrinotoxins will not be covered further by this review.

## 2. Venom Activities

As demonstrated by the plethora of symptoms possible from a single envenomation event, fish venoms conduct a multipronged attack against the vertebrate system. In congruence with this, studies have shown that the venoms target a variety of physiological systems and processes in order to deter potential predators.

### 2.1. Cardiovascular and Respiratory Systems

Deaths from fish envenomations have been attributed to cardiovascular effects such as severe hypotension and cardiac or respiratory failure [7,59,60]. This has led to much of the research being focused on this area. Cardiotoxicity from fish venoms was observed early on. When greater weeverfish, *Trachinus draco*, and lesser weever fish, *Trachinus vipera*, venom were introduced into five cats and one dog via IVs in femoral veins and a carotid artery respectively, effects on blood pressure were observed, including biphasic falls in arterial pressure and a biphasic rise and then fall in venous pressure. Ischemic injury patterns and rhythm disturbances on electrocardiograms were also observed [61]. Russell and Emery [61] also noted effects on respiration and that heart contractions continued in some animals following clinical death, a phenomenon that was later observed again in rabbits injected with venom from the lionfish *Pterois volitans* [62]. Skeie [60] found that injection of *T. draco* venom into mice led to marked vascular contraction, resulting in ischaemic necrosis and subsequent loss of ear and tail tips. Additionally, autopsies performed on animals killed by weever fish venom revealed frothy discharge in the respiratory system and a left ventricle devoid of blood, while the right side of the heart was always full [60]. Frothy, pink discharge in the respiratory-passages was also observed in cats, dogs, and rabbits injected with sublethal doses of California scorpionfish, *Scorpaena guttata*, venom [63].

Several common cardiovascular symptoms in response to fish envenomation are described throughout the literature [5,29,64,65]. These include changes in blood pressure and endothelium dependent smooth muscle relaxation, as well as inotropic and chronotropic responses. Fish venoms have elicited varying, often multi-phasic, blood pressure responses in experimental conditions (Table 1). In the case of the venoms of *T. draco* and the catfish *Arius thallasinus*, the steadily decreasing blood pressure resulted in the death of the animal models (a rabbit and a cat for *T. draco*, and rats and guinea pigs for *A. thallasinus*) [66,67]. Histaminergic receptors, muscarinic receptors, adrenoceptors, and leukotriene receptors have all been implicated in influencing the pressure responses to fish venom (Table 1). Carlson *et al.* [63] attributed the responses to *S. guttata* venom to the release of endogenous acetylcholine from muscarinic receptors. Similarly, Hopkins and Hodgson [68] found that the blood pressure response to the soldierfish, *Gymnapistes marmoratus*, venom was related to the release of endogenous nitric oxide from endothelial cells. Interestingly, a subsequent study showed that atropine was ineffective in changing the response, indicating a lack of muscarinic receptor involvement. Instead, the response was attenuated by SFAV, showing the proteinaceous nature of the bioactive components [64].

**Table 1:** Blood pressure effects caused by fish venoms under experimental conditions and the venom target causing these effects. A positive effect on blood pressure is indicated by a + sign and a negative effect by a − sign. Where the effect was multiphasic, the different phases are separated by a/in the order that they occurred.

Species	Pressure Effects	Model	Target	Source
***Arius thallasinus***	–	Rats, guinea pigs		Thulesius *et al.* [67]
***Trachinus draco***	−/+/−	Rabbit		Evans [66]
	−/+/−	Cats		Evans [66]
***Scatophagus argus***	−	Cat, guinea pig	Histaminergic receptor	Muhuri *et al.* [69]
***Pterois volitans***	+/−	Aneasthetised rats	Muscarinic receptors	Church and Hodgson [70]
	− (low dose), −/+ (high dose)	Anaesthetised rabbits		Saunders and Taylor [62]
***Scorpaena guttata***	Left ventricular end-diastolic pressure	Anaesthetized dog		Carlson *et al.* [63]
	+	Anaesthetized dog, pulmonary artery		Carlson *et al.* [63]
	−/+	Anaesthetized dog, systemic artery	Muscarinic receptors	Carlson *et al.* [63]
***Scorpaena plumieri***	+(low dose), +/− (lethal dose)	Anaesthetised rats	Adrenoceptors	Gomes *et al.* [71]
***Gymnapistes marmoratus***	−/+	Anaesthetised rat		Church and Hodgson [64]
***Synanceia horrida***	+	Anaesthetised rabbits	α1, β2 adrenoceptors and leukotriene receptors	Hopkins *et al.* [72]
	++/+/−	Anaesthetised rats	α1, β2 adrenoceptors and leukotriene receptors	Hopkins *et al.* [72]
	−	Rabbits		Saunders *et al.* [73]

Fish venoms have also been shown to cause endothelium-dependent relaxation in smooth muscle. Lyophilized *S. horrida* venom produced this response in the vascular smooth muscle of rats, which was abolished by atropine, indicating the involvement of muscarinic receptors. Interestingly the freshly milked venom, with a higher concentration of toxins, produced a biphasic response in which the initial relaxation was followed by sustained contraction [29]. The venoms of *G. marmoratus* and *P. volitans* were also found to produce endothelium dependent relaxation when introduced to precontracted pig coronary arteries. The mechanism of this relaxation went unexplored in *G. marmoratus* but it was found that, based on potentiation by atropine, *P. volitans* venom was acting at muscarinic receptors and causing relaxation through the release of nitric oxide (NO) as shown by inhibition with a nitric oxide synthase inhibitor [64,70]. A previous study on *G. marmoratus* venom also found that the relaxation response was most likely due to NO but via a muscarinic receptor independent mechanism [68]. In isolated rat hearts positive lusitropic effects were seen after exposure to both crude venom from the scorpionfish *Scorpaena plumieri* and from an isolated fraction with cardiovascular activity [71].

Changes in respiration and cardiac activity are often observed in response to fish venoms during experimental envenomation. Both *S. horrida* and *S. guttata* venoms have been shown to cause bronchoconstriction in animal models, a response that was attributed to the activity of released endogenous substance P at Neurokinin 1 (NK1) receptors in the case of *S. horrida* [72]. A study by Carlson *et al.* [63] found that *S. guttata* venom produced marked respiratory changes and even respiratory arrest in cats, rabbits, and dogs, as well as significant disruption of normal cardiac activity. Respiratory difficulties sometimes leading to respiratory arrest were also observed when the venom of *S. plumieri* was injected into anaesthetized rats [71]. Crude venom from *Scatophagus argus*, known as the spotted scat, slowed the respiratory rate in rats with no observed effects on respiratory amplitude [69]. In the same study, the opposite results were seen in cats, where respiratory amplitude was decreased though the rate remained unaffected. This hints at species selectivity. In anaesthetised rabbits, *P. volitans* venom caused increases in respiratory rate and cardiac disruptions, both of which were concentration dependent [62].

**Table 2:** Inotropic and chronotropic effects caused by fish venoms under experimental conditions and the apparent target. A positive effect is indicated by a + sign and a negative effect by a − sign. Where the effect was multiphasic, the phases are separated by a/in the order that they occurred.

Species	Inotropic Response	Chronotropic Response	Model	Target	Source
***Scatopagus argus***	−	−	Isolated guinea pig heart		Muhuri *et al.* [69]
	+	+	Isolated toad heart		Muhuri *et al.* [69]
***Pterois volitans***	−/+		Rat paced left atria	β1-adrenoceptors	Church and Hodgson [70]
		+	Rat spontaneously beating right atria	β1-adrenoceptors	Church and Hodgson [70]
	−	−	Isolated clam and frog heart		Cohen and Olek [74]
***Scorpaena guttata***	−/+	−/+	Rat atria	Muscarinic receptors and β-adrenoceptors	Carlson *et al.* [65]
***Gymnapistes marmoratus***	−/+	−/+	Isolated rat atria	Muscarinic receptors and β-adrenoceptors	Church and Hodgson [64], Hopkins and Hodgson [68]
***Scorpaena plumieri***		+/−	Anaesthetised rats		Gomes *et al.* [71]
	+	+	Isolated rat heart		Gomes *et al.* [71]
***Synanceia horrida***	−/+	+ (lyophilized venom), − (milked venom)	Isolated rat atria	M2 Muscarinic receptors and β1-adrenoceptors	Church and Hodgson [29]

As with blood pressure effects, the inotropic and chronotropic responses to fish venoms vary both on the fish species and the experimental animals selected (Table 2). Additionally, as with blood pressure responses, muscarinic receptors and adrenoceptors have been implicated in the cardiac changes seen in fish envenomations. A study on *S. guttata* venom found that the muscarinic receptors and adrenoceptors were moderating the cardiac responses via the release of endogenous acetylcholine and catecholamines respectively [65]. The study concluded that different venom components were initiating the release of the two endogenous stores. The cardiac responses to *S. plumieri*, *P. volitans*, and *G. marmoratus* venom were all attenuated by SFAV [50], once again demonstrating the involvement of proteinaceous venom components.

**Table 3:** Neuromuscular effects of fish venoms on various models and the apparent cause of these effects.

Species	Effect	Model	Apparent Cause	Source
***Tracinus draco***	Cell Depolarization	Rat brain particles	Increase in TTP^+^ outflow	Chhatwal and Dreyer [75]
***Scatophagus argus***	Postsynaptic blockage of electrically induced twitch response	Isolated chick biventer cervices preparation	-	Muhuri *et al.* [69]
	Relaxation response	Isolated rat duodenum preparation	-	Muhuri *et al.* [69]
	Contractile response	Isolated rat duodenum preparation, rat fundal strip, and rat uterus	-	Muhuri *et al.* [69]
***Gymnapistes marmoratus***	Contraction response	Guinea pig isolated ileum and longitudinal smooth muscle preparations	Released endogenous acetylcholine and cyclooxygenase metabolites acting at muscarinic receptors	Hopkins *et al.* [76]
	Contractile response	Chick biventer cervices muscle	Cell membrane pore formation	Church *et al.* [77]
***Pterois volitans***	Irregular muscular fibrillation and muscular blockade	Isolated neuromuscular preparation	Release of endogenous acetylcholine from presynaptic nerve terminal	Cohen and Olek [74]
	Contractile response	Chick biventer cervices muscle	Cell membrane pore formation	Church *et al.* [77]
***Synanceia horrida***	Contractile response	Guinea pig isolated ileum	Released endogenous substance P acting at NK1 receptor	Hopkins *et al.* [72]
	Reduced twitch height and increased basal tension	Chick biventer cervisis muscle preparation		Church and Hodgson [29]
	Contractile response	Chick biventer cervices muscle	Cell membrane pore formation	Church *et al.* [77]
***Synanceia verrucosa***	Cell Depolarization	Frog atrial heart muscle	Ca^+^ influx	Sauviat *et al.* [78]

### 2.2. Neuromuscular System

Envenomation symptoms such as paralysis, muscles spasms, and prolonged weakness clearly demonstrate that fish venoms target not only the cardiovascular system, but the neuromuscular system as well. Experimentally, fish venoms have been shown to elicit a number of other responses, including cell depolarization, muscle contraction (Table 3).

The TPP^+^ outflow in rat brain particles caused by *T. draco* venom was found to increase in a dose dependent manner. The presence of Ca^2+^ enhanced this depolarization activity but trypsinization and prolonged heat exposure of the venom abolished it, indicating a protein as the responsible agent [75]. The depolarization effect of reef stonefish, *Synanceia verrucosa,* crude venom (Table 3) resulted in the shortening of the action potential duration, an increase in peak tension at 2.9 µg/mL, a decrease in peak tension, as well as shortening of the plateau amplitude and duration at 5.7 µg/mL. The activity at 5.7 µg/mL also caused a shortening of the action potential repolarization and induced contracture in the muscle [78].

Mice injected with *P. volitans* venom in experimental conditions displayed skeletal muscular weakness indicative of neuromuscular activity [62]. This confirms the muscular blockade observed by Cohen and Olek [74], in neuromuscular preparation (Table 3).

Milked venom from *S. horrida* was deemed myotoxic based on the significant responses in chick biventer cervisis muscle preparation that it produced (Table 3). The fact that these responses were not observed when the experiments were performed with lyophilized venom points to the lability of the responsible component [29]. A study by Church *et al.* [77] expanded on this when it found that venom from *S. horrida*, along with venom from *P. volitans* and *G. marmoratus* produced contractile responses in chick biventer cervicis muscle, though these responses were attenuated by the removal of extracellular Ca^2+^. In cultured murine cortical neurones all three venoms increased intracellular Ca^2+^ in a dose-dependent manner, although to differing degrees. The mechanism of action appeared to be the Ca^2+^ independent formation of cationic pores in cell membranes which then allowed for the depolarization of the membranes via Ca^2+^ influx [77].

Based on the convulsions and paralysis observed from exposure to *S. argus* venom, it has been suggested that the venom is neurotoxic. However, studies have only confirmed its myotoxicity, as indicated by an increase in serum creatine kinase in mouse models following injection of the crude venom [79]. *S. argus* venom was also shown to work postsynaptically on isolated chick biventer cervices preparation to reversibly block the electrically induced twitch response, providing an insight into the mechanism by which paralysis occurs. However, the same study found that the venom had no effect on rat phrenic nerve diaphragm preparation pointing to species specific venom activity [69].

The venoms of the freshwater stingrays *Plesiotrygon iwame* and *Potamotrygon motoro* were also found to have myotoxic activity. At 24 h after the venoms had been injected into mouse skeletal muscle evidence of inflammation was observed, as well as coagulative necrosis. The rhabdomyolysis effect was more severe in those mice injected with the venom of *P. motoro* than those injected with *P. iwame* venom [80]. Rhabdomyolysis was also observed in a clinical case in which a fisherman was stung by a marine stingray, thought to be from the Dasyatis family [81].

Unlike with *S. argus*, neurotoxic activity was observed from a partially purified component of *A. thalassinus* venom. The neurotoxic activity was specific to acetylcholine receptors, where it blocked the transport of monovalent cations through the receptor channels [82].

### 2.3. Cytolytic Activity

Evidence indicates that nearly all fish venoms possess haemolytic activity. This is unsurprising, as haemolytic activity has also been found in the venoms of numerous other animals including snakes, jellyfish, sea anemones, bees, spiders, and scorpions. Like the haemolytic activity of other animal venoms, haemolysis caused by fish venom exhibits species sensitivity (Table 4). Venom from the catfish *Arius maculatus* is approximately four times more potent to chicken blood than to blood from sheep and humans [83]. *T. draco* venom was shown to be highly haemolytic towards rabbit erythrocytes, with an EC_50_ of 75 ng/mL, but was less haemolytic to erythrocytes from rats, and only slightly haemolytic those from mice and cattle [84]. Stonustoxin (SNTX), a lethal component from *S. horrida* venom was found to cause haemolysis of both rabbit and rat erythrocytes in a dose dependent manner, though rabbit cells were more susceptible [85]. *S horrida* crude venom has been shown to possess strong haemolytic activity against guinea pig erythrocytes and weaker activity against human and sheep erythrocytes [86]. A study looking at four closely related species of lionfish and two stonefish species showed that the haemolytic activity of each was highly selective for rabbit erythrocytes, and only *S. verrucosa* venom was able to produce weak haemolysis in guinea pig cells [51].

**Table 4:** Haemolytic activity of fish venoms on various animal erythrocytes. + = activity, X = no activity.

Species	Chicken	Sheep	Human	Rabbit	Rats	Mice	Cattle	Guinea Pig	Horse	Roach, Perch, Pigeon, Ox
***Arius maculatus***	+	+	+							
***Trachinus draco***	X	+	+	+	+	+	+	+	+	+
***Scatophagus argus***			+							
***Pterois antennata***	X	X		+			X	X	X	
***Pterois volitans***	X	X		+			X	X	X	
***Pterois lunulata***	X	X		+			X	X	X	
***Dendrochirus zebra***	X	X		+			X	X	X	
***Hypodytes rubripinnis***				+						
***Scorpaena guttata***		+								
***Notesthes robusta***			+							
***Inimicus japonicus***	X	X		+			X	X	X	
***Synanceia horrida***		+	+	+	+			+		
***Synanceia verrucosa***	X	X		+			X	+	X	

The results from Shiomi *et al.* [51] show that the haemolytic activity against rabbit erythrocytes ranged widely among the six species, from 740 haemolytic units mg^−1^ venom for the lionfish *Pterois antennata* to 23,700 haemolytic units mg^−1^ venom for *P. volitans*. Similarly, a study on the crude venom of the lionfish *Pterois lunulata*, bearded ghoul *Inimicus japonicus*, and red velvetfish *Hypodytes rubripinnis* found that all three were haemolytic to rabbit erythrocytes, but *P. lunulata* had 10 fold higher activity than *I. japonicus* and 100 fold higher activity that *H. rubripinnis* [87].

In a few animal venoms, phospholipase A_2_ (PLA_2_) contributes to the cytotoxic activity [88,89,90]. However, studies consistently demonstrate a complete lack of PLA_2_ proteins in piscine venoms. Instead, haemolysis caused by fish venoms must use alternative mechanisms. In *S. argus* the mechanism used appears to be heat sensitive and calcium dependent [91].

In addition to their haemolytic activity, fish venoms also possess the ability to lyse other cell types. *S. argus* venom has been found to cause the lysis of HeLa cells and platelets [79]. The venoms of the toadfish, *Thalassophryne nattereri*, and *S. horrida* both possess platelet-lysing activities as well, but *S. horrida* venom does not cause significant lysis of HeLa cells [92,93]. Endothelial cells have been shown to lyse in a dose-dependent manner when exposed to crude *T. nattereri* venom *in vitro*, as have C2C12 murine myoblasts [42,92].

### 2.4. Enzymatic Activity

Enzymatic activity is common in animal venoms, which is unsurprising as it is instrumental in the deleterious breakdown of physiological structures. Enzymes may cause damage in their own right, or may work as spreading factors for the other venom toxins. In fish venoms, it has even been suggested that proteolytic enzymes could be partially responsible for the extreme lability of the other venom components [13]. Unsurprisingly, proteolytic activity has been confirmed in the venoms of many fish species (Table 5).

**Table 5:** Fish venoms that have been found to exhibit proteolytic activity against casein, gelatin, and fibrinogen (+ = activity, X = no activity).

Species	Casein	Gelatin	Fibrinogen
***Dasyatis guttata***	+	+	+
***Potamotrygon falkneri***	+	+	+
***Potamotrygon henlei***		+	
***Potamotrygon scobina***	+		
***Potamotrygon orbygnyi***	+		
***Plesiotrygon iwamae***		+	
***Arius thallasinus***	+		
***Arius maculatus***	+	+	
***Thalassophryne nattereri***	+	+	
***Thalassophryne maculosa***	+		
***Scatophagus argus***	+		
***Scorpaena plumieri***	+	+	
***Pterois volitans***		+	
***Notesthes robusta***		+	
***Synanceia horrida***	X	X	

*S. verrucosa* venom was shown to have 10 different peptidase activities [94]. In a study comparing the sting tissue extract of the marine stingray *D. guttata* and the fluvial stingray *P. falkneri*, it was found that both contained proteolytic enzymes against casein, gelatin, and fibrinogen, although the molecular weights of the enzymes differed between the two species [95]. When crude *P. henlei* venom was subjected to SDS-PAGE, several of the resulting bands showed hydrolytic action against gelatin [96], and gelatinolytic activity was also found in the venom of *P. iwamae* [97]. Both *P. scobina* and *P. orbygnyi* crude venoms were shown to have low levels of proteolytic activity against casein [98], a result that was also found with the toadfish *T. nattereri* and *T. maculosa* [99,100], the catfish *A. thallasinus* [67], and the butterfish *S. argus* [91]. Additionally, *T. nattereri* venom was shown to have gelatinolytic activity [101]. Like *P. henlei*, the venoms of both *N. robusta* and *P. volitans* were found to contain proteases acting against gelatin using SDS-PAGE methods [52,102]. Additionally, the protease in *P. volitans* venom was found to have a molecular weight around 45 kDa. A similar zymography experiment showed that the catfish *A. maculatus* also had gelatinolytic components between 43 kDa and 97 kDa [83]. The venom of *S. plumieri* was found to have proteolytic activity against both casein and gelatin [13]. Furthermore the proteolytic enzymes were shown to be very stable, which was hypothesized to indicate the presence of a zymogen. A 72 kDa gelatinase was isolated from the *S. plumieri* venom, although attempts to sequence the *N*-terminus were unsuccessful, indicating that it was most likely blocked, which could be related to the holocrine-type secretion used by venomous fish [13]. *S. horrida* venom lacked activity against both casein and gelatin [103]. It is possible that venom processing prior to experimentation was responsible for a loss in proteolytic activity, though Khoo *et al.* [93] also found that the venom lacked activity against casein.

Fish venoms have also been shown to contain a number of enzymes other than proteases. *S. argus* venom has both alkaline and acid phosphatase activity, as well as phosphodiesterase activity [104]. Both *G. marmoratus* and *S. horrida* also showed evidence for all of these enzymes, as well as esterase [105]. The levels of alkaline and acid phosphatase found in *S. argus* venom were higher than those found in either *G. marmoratus* or *S. horrida* venom, but *S. horrida* had substantially less phosphodiesterase activity than the other two species that have comparable phosphodiesterase activity. Esterase and alkaline phosphatase activity were also found in *A. thalassinus* venom but at levels significantly lower than those in *G. marmoratus* [67]. Garnier *et al.* [94] found that *S. verrucosa* venom had activity for eight different esterases targeting various substrates. Additionally, angiotensin-converting enzyme activity was found in *T. nattereri* venom, where it contributes to the venom’s inflammatory response [106].

Hyaluronidase is a common venom enzyme and facilitates the distribution of toxic components by breaking down the structurally important hyaluronan around the envenomation site. In keeping with this, hyaluronidase activity has been found in several different fish venoms. These include stonefish, soldierfish, lionfish, weeverfish, and stingrays [60,94,95,105,107,108,109]. Even though *N. robusta* is closely related to stonefish, soldierfish, and lionfish, when the crude *N. robusta* venom was tested for hyaluronidase activity, none was detected [52].

As previously stated, no fish venoms have been found to exhibit PLA_2_ activity. However, *S. argus* was recently shown to possess phospholipase C activity [91]. Just as PLA_2_ causes haemolysis in a some terrestrial venoms, Ghafari *et al.* [91] hypothesized that PLC could be a haemolytic agent in *S. argus* venom.

### 2.5. Nociceptive, Edematic, and Necrotic Activities

Intense pain and severe edema are the major symptoms common to the vast majority of fish envenomation. The two can be related, as shown by studies on the venoms of *Potamotrygon* stingray species. Immediately after venom injection, neuropathic pain is observed, but the pain seems to lessen during the subsequent inflammatory period, indicating that the second phase of pain involved the inflammatory processes of leukocyte rolling and adhesion [96,98]. Similar studies have confirmed that venom from several different fish species causes severe pain in mouse models [50,87,110,111]. A single pain-causing protein fraction was isolated from *N. robusta* venom, and attributed as the major source of nociceptive action for the crude venom, but its mechanism of action remains unknown [52]. When the venom of the toadfish *T. nattereri* venom was split into several fractions, based on observed peaks from cation-exchange chromatography it exhibited nociceptive properties in each of these fractions [112]. The nociceptive activity was attenuated by an opioid analgesic and theorized to possibly be due to both direct toxicity and via kinin due to the venom’s tissue-kallikrein-like activity [49,112]. Despite the fact that extreme pain is the major symptom of fish envenomation, it has been suggested that both *S. argus* and *P. volitans* venom contain antinociceptive compounds [102,104]. In both cases this was based on the fact that venom caused a dose-dependent increase in the activity of Na^+^, K^+^, and ATPase, which are known to mediate pain. These studies add to the understanding of the nociceptive properties of fish venoms. However, much remains to be discovered in order to truly understand this facet of venom activity.

The literature has focused more on the inflammation and edema that often accompany extreme pain during envenomation events. As with nociceptive activity, kinins were indicated in playing a major role in the inflammatory process of *T. nattereri* [112]. The kallikrein-kinin pathway was also implicated in the inflammatory action brought on by injection of *S. plumieri* venom in mice, although venom did not cause a direct release of kinin [113]. However, the venom of the toadfish *Porichthys porosissimus* caused an inflammatory response that involved the more classic cellular recruitment of neutrophils that proceeded macrophages and rolling leukocytes [110].

Similarly, a study examining the inflammatory actions caused by the venom of the toadfish *Thalassophryne maculosa* revealed that it first caused recruitment of mononuclear cells and then induced a delayed increase in recruitment of neutrophils [114]. *T. maculosa* venom was previously found to induce immediate edema in mice, which subsequently resulted in necrosis within 48 h due to venular stasis and arteriolar contraction [100].

Interestingly, where *T. maculosa* venom was found to cause venular stasis [100], *T. nattereri* venom was shown to cause arteriolar stasis, along with increased vascular permeability and thrombosis [92]. Parejo-Santos *et al.* [115] showed that the venom altered the extracellular matrix in such a way as to create an inhospitable environment for the inflammatory cells that would promote healing, thus explaining the chronic, slow healing injuries that result from *T. nattereri* stings and can lead to necrosis. Representative drugs for both steroidal and non-steroidal anit-inflammatory agents failed to reduce edema from *T. nattereri* venom, as did inhibitors of serotonin and nitric oxide synthase [49].

A study on the lung damage caused by *S. plumieri* venom in mice found that venom injected into the footpad or peritoneal cavity led to venom deposition in the lung. This caused alveolar edema along with neutrophil recruitment and IL-6 production, subsequently leading to apoptosis, lung injury and hemorrhage [116]. Mice injected with *P. volitans* venom also showed evidence of edema and hemorrhage in lung tissues. Additionally, edema of the heart and brain parenchyma was found, along with cloudy swelling and hemorrhaging in the renal tubules [102]. *S. argus* venom was also found to cause cloudy swelling in renal tubules following intraperitoneal injection in mice [111]. Additionally, *S. argus* venom produced hemorrhage in the stomachs of mice that rabbit antiserum could not attenuate pointing to the non-proteinaceous nature of the responsible component [117]. The study also found that edema induced by *S. argus* venom in mice could last for the whole entire 72 h of observation post-injection. A second study on *S. argus* venom found that the edema was likely histamine independent [79], indicating that other inflammatory mediators were at work.

Venom from the catfish *Pseudoplatystoma fasciatum* was found to produce severe inflammation in mice. As inhibitors for COX-2 and cytokines, as well as a 5-HT receptor antagonist attenuated the response, it was concluded that leukotriene, prostaglandin, and serotonin were all involved in the inflammatory response [118].

Similar to teleost fishes, such as *P. lunulata*, *I. japonicus* and *H. rubripinnis* [87], stingray venoms also lead to acute edema. Edema was found to be dose-dependent with venom from the fluvial species *Potamotrygon scobina* and *Potamotrygon orbignyi.* The study also found that incubating the venom at high temperatures attenuated the edema response [98]. *Potamotrygon henlei* venom triggered a significant edematic response in mice paws. In the same study, neutrophil and macrophage recruitment, increases in interleukin levels and increased vascular permeability were all observed in mouse models, providing a picture of the inflammatory response to the venom [96]. *P. motoro* venom was found to cause sustained edema for up to 48 h post injection in mice. Again macrophage and neutrophil increases were observed at the injection site, as well as increases in lymphocytes and eosinophil [119]. Kimura *et al.* [119] also found that the venom caused mast cell degranulation and altered the epidermal base layer. Interestingly, spine extracts from *Potamotrygon falkneri* produced only mild inflammation in mice, but did produce tissue necrosis as early as 3 hours post injection [97].

### 2.6. Immune System Modulation

Some work has been done on the ability of fish venom, specifically that of *T. nattereri,* to modulate the immune system. On top of mediating inflammation, macrophages also allow for intrinsic plasticity of the innate immune response to environmental signaling and for the modulation of acquired immunity. The venom of *T. nattereri* was found to modulate the kinetics of leukocyte influx in mouse injured footpads, which impaired the transit of neutrophils and affected macrophage survival, a combination that led to a deficient healing phase [120]. Nattectin, a C-type lectin found in *T. nattereri* venom, was shown to lead to M1 reprogramming of macrophages, which manifested via an increase in the amount of MHC-II-dependent Ag present. This activity was found to be dependent on the lectin function of Th1 cytokines and the molecular complex of migration that allows them access to secondary lymphoid organs [121]. A subsequent study found that nattectin not only activates innate immune macrophages thereby inducing typical dendritic cell functions, but also drives T-cell responses to the Th1 phenotype [122].

Long-term immune protection requires the persistence of vaccine antibodies and/or the generation of immune memory cells capable of rapid and effective re-activation upon subsequent exposure. Data from work on *T. nattereri* venom in mice strongly implies that IL-17A derived from effector memory T-cells govern the differentiation of germinal center derived-memory-B cells into antibody-secreting cells and maintain their longevity through a mechanism directly dependent on B-cell receptors, and *T. nattereri* venom was found to affect these processes [123,124,125]. Additionally, it was found that the proteolytic activity of a novel toxin family from *T. nattereri* venom, known as Natterins, is critical for the hierarchical differentiation of antibody-secreting cells, and for the adjuvanticity of the venom [126,127]. Overall, the data gained from experiments on immune responses to *T. nattereri* venom support the established concept that generation of vaccine-induced TH17 cells and IL-17 production are crucial for immunological protection and demonstrate the use of venom toxins as physiological tools.

## 3. Venom Components

Despite the relative lack of research focused on fish venom toxins, a number of protein components have been isolated and partially characterized. Much of the cardiovascular, neuromuscular, and cytolytic activities in some fish venom can be linked back to a single proteinaceous agent, indicating that piscine venoms may be less complex than those of terrestrial vertebrates [5]. However, it is possible that studies thus far have underestimated the true diversity of venom components because of venom lability and the incompatibility of some components with experimental conditions; in particular the use of gel analyses of whole venom in the early literature while effective for observing proteinaceous components above 10 kDa, are unable to show the smaller molecular weight components. Fish venoms have been found to contain various enzymes, along with some non-proteinaceous components. Additionally, there is a high probability that there are small, bioactive peptides present in the venom that have been overlooked in studies thus far.

### 3.1. Proteinaceous Toxins

There have been a number of toxins isolated and characterized from a range of fish venoms to date (Table 6). In some cases, these are quite similar to each other despite belonging to different species or clades.

**Table 6:** Toxins found in fish venoms to date, as well as their molecular weight.

Species	Toxin	MW	Source
***Synanceia horrida (trachynis)***	Trachynilysin (TLY)	158 kDa (2 subunits)	Colasante *et al.* [128]
	Stonustoxin (SNTX)	148 kDa (2 subunits)	Poh *et al.* [31]
***Synanceia verrucosa***	Verrucotoxin (VTX)	322 kDa (4 subunits)	Garnier *et al.* [94]
	Neoverrucotoxin (neoVTX)	166 kDa (2 subunits)	Ueda *et al.* [129]
	Cardioleputin	46 kDa	Abe *et al.* [130]
***Notesthes robusta***	Nocitoxin	169.8–174.5 kDa	Hahn and O’Connor [52]
***Pterois volitans***	*	2 subunits, both ~75 kDa	Kiriake and Shiomi [131]
***Pterois antennata***	*	2 subunits, both ~75 kDa	Kiriake and Shiomi [131]
***Pterois lunulata***	*	160 kDa (2 subunits)	Kiriake *et al.* [87]
***Inimicus japonicus***	*	160 kDa (2 subunits)	Kiriake *et al.* [87]
***Hypodytes rubripinnis***	*	160 kDa (2 subunits)	Kiriake et al. [87]
	Karatoxin	110 kDa (2 subunits)	Nagasaka et al. [132]
***Sebastapistes stongia***	*	N/A	Chuang and Shiao [133]
***Scorpaenopsis oxycephala***	*	N/A	Chuang and Shiao [133]
***Sebasticus marmoratus***	*	N/A	Chaung and Shiao [133]
***Dendrochirus zebra***	*	N/A	Chaung and Shiao [133]
***Scorpaena plumieri***	Sp-CTx	121 kDa (2 subunits)	Andrich *et al.* [134]
	Plumieribetin	14 kDa	Evangelista *et al.* [135]
	SP-CL 1-5	16.8–17 kDa	Andrich *et al.* [136]
***Trachinus draco***	Dracotoxin	105 kDa	Chhatwal and Dreyer [75]
***Trachinus vipera***	Trachinine	324 kDa (4 subunits)	Perriere *et al.* [137]
***Scatophagus argus***	SA-HT	18 kDa	Karmakar *et al.* [138]
***Thalassophryne maculosa***	TmC4-47.2	Unknown	Sosa-Rosales *et al.* [100]
***Thalassophryne nattereri***	Nattectin	15 kDa	Lopes-Ferreira *et al.* [101]
***Plotosus canius***	Toxin-PC	15 kDa	Auddy *et al.* [139]
***Cathrops spixii***	Wap65	54 kDa	Ramos *et al.* [58]

* Unnamed stonefish toxin-like toxin (based on similarities to SNTX and VTX).

**Figure 3:**
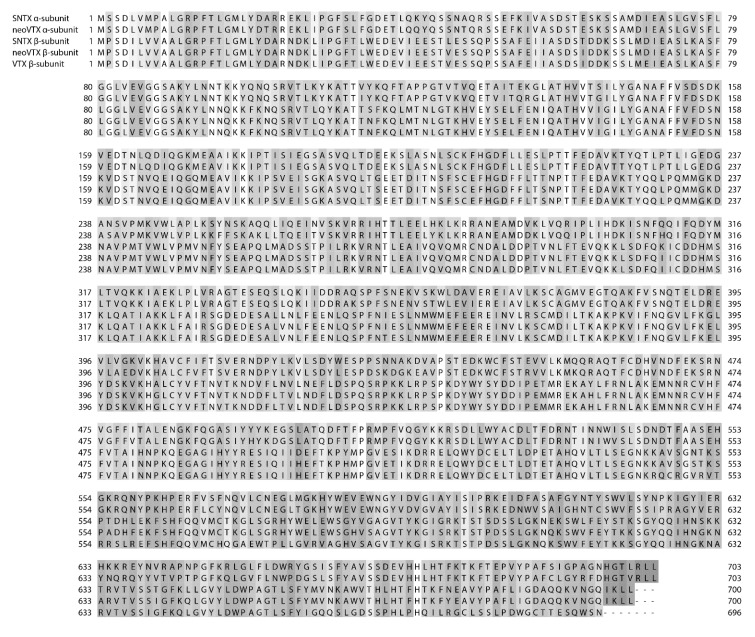
Sequence alignment of the α-subunits of SNTX and neoVTX, and the β-subunits of SNTX, neoVTX, and VTX (Uniprot accessions numbers Q98989, A0ZSKA, Q91453, A0ZSK4, and Q98993 respectively). Amino acid numbers are shown on both the left and right, and sequence discrepancies are highlighted in grey in a gradient based on similarity.

Verrucotoxin (VTX), isolated from *S. verrucosa* venom is a tetrameric protein with a total mass of 322 kDa, composed of two glycosylated α-subunits and two glycosylated β-subunits. It has lethal and haemolytic activities and causes hypotension and cardiac arrest when injected into mice [94]. Though VTX exhibits the extreme lability recognised in fish venoms, Garnier *et al.* [140] were able to isolate it in complex with stabilizing proteins (p-VTX). Both VTX and p-VTX were found to exert negative inotropic and chronotropic effects on frog atrial heart muscle via the inhibition of Ca channels and the opening of K channels [140]. A more in-depth study on VTX concluded that the toxin acts as a β_1_-adrenoceptor agonist via the cAMP-PKA pathway, which then leads to an increase in L-type Ca^2+^ currents [141].

A second toxin was isolated from *S. verrucosa* venom and shown to be a haemolytic dimer composed of α and β subunits. This 166 kDa toxin was named neoVerrucotoxin (neoVTX) and was inhibited by anionic lipids. The α and β subunits were shown to have as high as 49% sequence identity with each other and are linked by non-covalent bonds as opposed to disulfide bridges, even though neoVTX contains 18 cysteine residues [129]. Based on the finding of Ueda *et al.* [129] that neoVTX contains 10 free thiol groups, this is not entirely surprising.

NeoVTX was found to be highly similar to stonustoxin (SNTX), which had been previously isolated from *S. horrida* venom. SNTX is also a dimeric protein composed of an α and a β subunit, and has a native molecular weight of 148 kDa [31]. The β-subunit of neoVTX showed a 95% homology with the β-subunit of SNTX (Figure 3) as opposed to only a 90% sequence homology with the β-subunit of VTX, and the α subunits of neoVTX and SNTX show 87% identity [129]. Interestingly, the β-subunit of VTX was found to have 96% sequence identity with the β-subunit of SNTX (Figure 3), indicating that SNTX may be more closely related to both the *S. verrucosa* toxins than they are to each other [142]. Additionally, both SNTX and VTX are predicted to have amphipathic α-helical wheels [142,143].

Other similarities between SNTX and neoVTX are that non-covalent bonds hold the two subunits of SNTX together, instead of disulfide bridges, and that neither are glycosylated [129,144]. Additionally, it was found that there is a 50% sequence identity between the α and β subunits of SNTX [143]. However, the two toxins differ in their cysteine content. SNTX has only 15 cysteine residues, and 5 free thiol groups, indicating that the other 10 are involved in intrachain disulfide bonds [144].

SNTX demonstrates haemolytic activity. Similarly to crude venom, it lysed rat, guinea pig, and rabbit erythrocytes at different concentrations, but was ineffective against human and mouse erythrocytes [31]. An osmotic protectant assay using rat erythrocytes showed that the haemolytic activity of SNTX was due to the formation of pores, approximately 3.2 nm in diameter, in erythrocyte membranes [145]. This haemolytic action subsequently induces platelet aggregation in whole blood from affected species [85]. SNTX also demonstrates edematic properties and potent lethal activity with an LD_50_ in mice of 0.017 μg/g [31]. Studies found that the toxin’s cationic lysine and arginine residues play a large role in mediating both haemolytic and lethal activity [145,146]. Haemolytic and lethal activities were abolished when 5,5'-dithiobis(2-nitrobenzoic acid) was introduced in order to modify the free thiol groups. However, the introduction of dithiothreitol to reduce the disulfide bonds in the protein and abolish its tertiary structure, actually potentiated the toxin’s activity [144]. This shows that, surprisingly, while the free thiol groups of SNTX are important for moderating activity, the intrachain disulfide bonds are not. Interestingly, monoclonal antibodies raised against SNTX were able to neutralize the lethal but not haemolytic activity, indicating that two separate domains are responsible for these activities [147]. In anaesthetised rats SNTX had negligible effect on skeletal muscle contractility but induced marked hypotension and endothelium-dependent vasorelaxation [59]. The study found that vasorelaxation was mediated by the release of either endogenous nitric-oxide (NO) or a NO releasing substance. A subsequent study found that endogenous hydrogen sulfide works synergistically with NO to cause SNTX induced-vasodilation [148].

Trachynilysin (TLY) is another dimeric protein isolated from *S. horrida* venom. It too is composed of α (76 kDa) and β (83 kDa) subunits [128], but has not been investigated for haemolytic or lethal activity. Instead, Colasante *et al.* [128] showed that TLY acted presynaptically on frog motor nerve terminals to deplete small clear synaptic vesicles while leaving large dense-core vesicles unaffected. The result was supported by a second study that found TLY to reduce contractile force in frog atrial heart muscle due to endogenous acetylcholine being released and acting at muscarinic receptors [149]. The same study found TLY to increase the Ca^2+^ entering the cell. Another study showed that TLY could cause exocytosis of large dense-core vesicles from chromaffin cells, but only when there was extracellular Ca^2+^ present [150]. Just as with SNTX, TLY was found to cause the formation of cationic pores in cell membranes through irreversible membrane insertion [151].

Large toxins like those from the stonefish have been found in several other scorpaeniform species as well. The crude venoms from two lionfish species, *P. volitans* and *P. antennata* were found to contain large dimeric proteins that cross-reacted with antibodies raised against neoVTX and had high sequence homology with stonefish toxins [131]. Similar toxins were also found in the venoms of *P. lunulata*, *I. japonicus*, and *H. rubripinnis* using cDNA analysis [87]. The sequencing of these five toxins, as well as SNTX and neoVTX, has shown that they each possess B30.2/SPRY domains, which are thought to aid protein-protein interactions [87,129,131]. A recent study looking at genetic data from four species of scorpionfishes predicted that all four would have large toxins similar to those found in the other scorpaenoids. *Sebastapistes stongia*, *Scorpaenopsis oxycephala*, *Sebastiscus marmoratus*, and *Dendrochirus zebra* were all found to contain genes coding for large heterodimeric toxins that maintained high sequence homologies with those found in stonefish and lionfish [133]. Chuang and Shiao [133] also found that the genes were undergoing negative selection.

A dimeric glycoprotein of 121 kDa was isolated from *S. plumieri* and found to have both haemolytic activity and biphasic vasoactivity, the latter of which involved NO as a mediator [134]. Called Sp-CTx, the protein’s mechanism of action was found, using osmotic protectants, to be pore formation in cell membranes. In addition to forming pores like stonefish toxins, Sp-CTx peptide fragments aligned with sequences from neoVTX, SNTX, and the toxins from both *P. volitans* and *P. antennata* [152]. Interestingly, Gomes *et al.* [152] also found that Sp-CTx forms molecular aggregates, and predicted that the number of units per aggregate could predict the diameter of the pore formed by the toxin.

Based on the highly homologous primary structures of the large scorpaenoid toxins, it is reasonable to surmise that they also share a mechanism of action. This is supported by the fact that SNTX, trachynilysin, and Sp-CTx have all been shown to work by forming pores in cell membranes. Since the toxins appear to all have a homologous function, it stands to reason that the pharmacology exhibited by one may also apply to the others.

A number of other protein toxins have been found in scorpaenoid venoms as well, although the information available for them is limited. Nocitoxin, the pain-causing protein from *N. robusta* was found to be the only protein present in a nociceptive fraction of a peak from Sephacryl S-200 chromatography. It was also found to be monomeric with only a single band present around 170 kDa on an SDS-PAGE gel after denaturization. In addition to nociceptive activity, nocitoxin was also found to have slight haemolytic activity against human erythrocytes [52]. Cardioleputin, a 46 kDa protein from *S. verrucosa* that was demonstrated to have inotropic and chronotropic effects on guinea pig atria [130]. Amino acid analysis suggested that no cysteine residues were present. Karatoxin was isolated from *H. rubripinnis* venom using a combination of chromatography methods [132]. Karatoxin is a 110 kDa complex that acts as a D-mannose binding lectin. Although it lacks haemolytic activity karatoxin has been shown to have cytolytic, mitogenic, and chemotactic effects, as well as agglutinating effects on rabbit erythrocytes [132,153]. Lectins have also been found in the venom of *S. plumieri*. A 14 kDa B-type lectin named Plumieribetin was isolated and found to inhibit α1β1 integrin [135]. Interestingly, the protein appeared to oligomerize with no loss of activity in an inhibition ELISA. A group of five isolectins (SP-1-5) were found in an agglutinating fraction of *S. plumieri* venom [136]. One of these isolectins was sequenced and found to have homology with fish C-type lectins.

In addition to the Scorpaenoides, proteinaceous toxins have been isolated from other taxonomic groups of venomous fish. The major toxic component of the greater weeverfish, *T. draco*, was isolated and found to be a 105 kDa protein named “dracotoxin.” As with *T. draco* crude venom, dracotoxin showed high specificity to rabbit erythrocytes, with an EC_50_ of 3 ng/mL, as compared to an EC_50_ of 7.5 ng/mL for crude venom, and little to no haemolytic activity against other animal erythrocytes [75,84]. The study found that Dracotoxin caused haemolysis via membrane depolarization from interactions with membrane glycophorin [75]. However the toxin was not tested for other bioactivities. A toxin has also been isolated from the closely related lesser weeverfish *T. vipera*. Named “trachinine,” this toxin represents a lethal fraction from the venom, has a molecular weight of 324 kDa, and appears to be composed of four identical subunits of 81 kDa each. The toxin was found to kill 20 g male mice instantly at iv doses of 2–2.5 μg, but further activity was not investigated [137]. It is unclear as to whether these toxins are related to those found in scorpaeniformes, though it is possible, as they appear to have similar activities and structural characteristics.

The only toxin isolated from *S. argus* venom so far, SA-HT, is relatively small. It is an 18 kDa protein with severe haemorrhagic activity to stomach walls but lacking in haemorrahagic activity to cutaneous tissue [138]. The study also found that SA-HT produced dose-dependent edema, capillary permeability, muscle contraction or relaxation, mast cell degranulation, and increased levels of plasmin and malonaldehyde in various animal models.

A 15 kDa myotoxic polypeptide called TmC4-47.2 was found in *T. maculosa* venom and appeared be selective for skeletal muscle, increasing miniature endplate potentials and causing depolarization on frog neuromuscular junctions [100]. The 15 kDa toxin Nattectin was isolated from the venom of *T. nattereri*, which is closely related to *T. maculosa*. However Nattectin, was shown to be a galactose-specific lectin that caused agglutination of human red blood cells and induced neutrophil mobilization in mice in a Ca^2+^ independent manner [101]. Additionally nattectin was actually shown to improve integrin-mediated cell adhesion in HeLa cells and improve their resistance to apoptosis [154], as well as the immune system effects previously discussed.

A Warm Temperature Acclimation-Related Protein 65-kDa (Wap65) was discovered in the sting venom of *C. spixii* and found to have pro-inflammatory action, by increasing the number of rolling leukocytes [58]. Another catfish toxin, the 15 kDa protein Toxin-PC, was isolated from *Plotosus canius* and found to have non-competitive neuromuscular blocking activity in cardiac tissues via interactions with K^+^ channels [139].

### 3.2. Enzymes

*T. nattereri* venom was found to contain a tissue-kallikrein-like enzyme that caused the release of products that played only a minor role in venom-induced inflammation. However it was found that these products played a role in nociception and edema caused by the venom [49]. This was affirmed later when the main toxic components of *T. nattereri* venom (18.8% of the total venom gland transcript) were found to contain the 5 proteins called Natterins that form a novel toxin family [112,155]. The Natterins range in size from 41.4 kDa (Natterin 4, 387 amino acids) to 5.9 kDa (Natterin P, 71 amino acids). They have kallikrein activity, and are also allodynic and edema inducing [112]. Natterins cleave type I and type IV collagen, leading to necrosis of the affected cells [154]. Recently, it was found that Natterins inhibit interactions between leukocytes and the endothelium, and reduce neutrophil accumulation, thereby achieving some anti-inflammatory effects that contradict their edematic activity. These effects were found to be dependent on negative signals derived from the TLR2-TLR4/Myd88 signaling cascade that is mediated by the activation of serine/threonine phosphatases and key signaling of the PI3K molecule [156]. Interestingly, it appears that the Natterin family is undergoing accelerated evolution [112], a phenomenon that is more often associated with predatory toxins.

**Figure 4:**
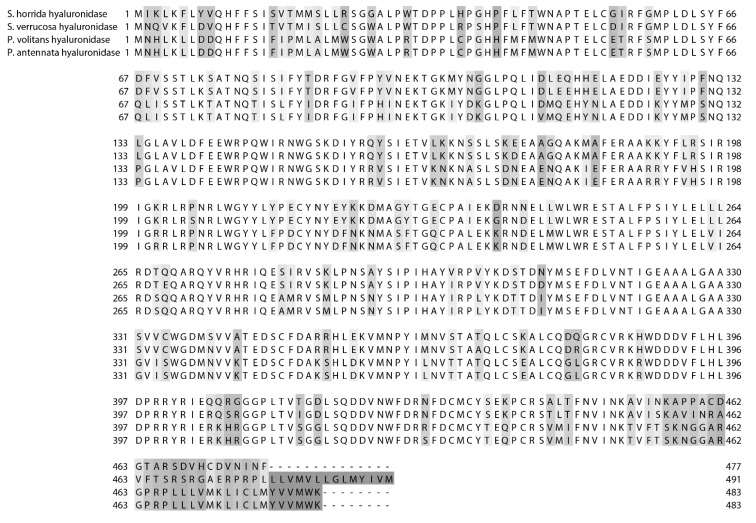
Sequence alignment of the hyaluronidases from *S horrida*, *S. verrucosa*, *P. volitans*, and *P. antennata* (Uniprot accessions numbers Q801Z8, E5RWZ0, K7ZMF5, and K7ZPU7 respectively). Amino acid numbers are shown on both the left and right, and sequence discrepancies are highlighted in grey in a gradient based on similarity.

Several hyaluronidases have been isolated and characterized from fish venoms A hyaluronidase from *S. horrida* venom was first isolated by Poh *et al.* [157] and found to have a molecular weight of 62 kDa. Later, it was named SFHYA1, and amino acid sequencing revealed that it contains three *N*-glycosylation sites [158]. SFHYA1 was found to be specific for hyaluronan [159]. Interestingly, it appeared to be most closely related, though with less than 50% identity [160] to the sperm-surface PH-20 family of hyaluronidases which have a wider range of substrates [158]. A second hyaluronidase was isolated from the freshwater stingray *P. motoro*, and was found to have an optimal pH of 4.2, a molecular weight of approximately 79 kDa, and a relatively high affinity for hyaluronan as a substrate [109]. A hyaluronidase was also found in *S. verrucosa* venom. It was only partially purified but the primary sequence was determined using cDNA data. It shared less than 50% identity with other animal hyaluronidases but a 92% sequence identity with SFHYA1, including the same three glycosylation sites, which is not entirely surprising considering their close taxonomic relationship [160]. The study also demonstrated experimentally the enzyme’s role as a venom ‘spreading factor’ and found it to have a molecular weight of 59 kDa and an optimal pH of 6.6. Hyaluronidases from the lionfishes *P. volitans* and *P. antennata* were also found to be neutrally active with optimal pHs of 6.6 and selective against hyaluronan. Additionally, they had 99.6% sequence homology to each other and 72%–77% homology with the stonefish hyaluronidases (Figure 4).

The lionfish hyaluronidase sequences show five possible glycosylation sites, including the three found in both stonefish hyaluronidases [108]. Based on this evidence, it is reasonable to theorize that the homology of fish venom hyaluronidases can be predicted based upon the taxonomic relationship of the fish species from which they come. In addition, as one would imagine, fish venom hyaluronidases are more closely related to each other than to other animal hyaluronidases.

### 3.3. Bioactive Peptides

One study on the venom of the catfish *C. spixii* found that the venom contained nearly 50 peptides under 3000 Da using RP-HPLC and MALDI-ToF analysis [58]. These peptides were divided into five different portions after RP-HPLC fractionation and were not individually purified. Additionally, the structural characteristics of the peptides were not examined. It was shown that two of the venom peptide fractions induced an increase in the number of rolling leukocytes, and the other three fractions induced venular stasis. Additionally, one of these fractions produced a strong, irreversible arteriolar contraction. Protein fractions obtained from the sting venom were unable to elicit these results, indicating that the peptides and not protein components were responsible for tissue damage caused by the venom. Two of the peptide fractions also showed antimicrobial effects.

Two small, bioactive peptides have been isolated from the venom of the stingray *P. orbignyi*. The first, named Orpotrin, was found to have a molecular mass of 1001.49 Da and a primary structure of HGGYKPTDK via *de novo* ms-ms sequencing [161]. The study found that the sequence aligned with the 97–105 residues of creatine kinase but lacked similarities to known bioactive peptides. Orpotrin was found to elicit vasoconstrictive effects on mouse creamaster muscle in physiological conditions when 20 μL of 1 mM of the peptide was topically applied. The action appeared to be selective and direct on the large arterioles of the microcirculatory network, however the mechanism of action was not elucidated.

The second bioactive peptide from *P. orbignyi* was named Porflan, had an amino acid sequence of ESIVRPPPVEAKVEETPE and molecular weight of 2006.09 Da, and did not show any homology with known peptides and proteins [162]. The peptide was shown to be pro-inflammatory by increasing rolling leukocyte numbers in mouse creamaster muscle post-capillary venules. Additionally, molecular dynamic simulations predicted that Porflan is unable to cross biological membranes unassisted and therefor may target active transport and extracellular domains to elicit activity.

A 7.6 kDa peptide from *P. volitans* venom was isolated and tested for its effectiveness against cancer cells [163]. Although the structure of the peptide was not elucidated, it was found that it selectively induced apoptosis in HEp2 and HeLa cells *in vitro* while having no deleterious effects on human lymphocytes. The exact mechanism of action by which the peptide causes apoptosis was not examined.

### 3.4. Non-proteinaceous Components

Though proteinaceous toxins and enzymes appear to be the major components in piscine venoms, there is evidence that they contain non-proteinaceous active compounds. For example, a dialyzable fraction from *T. vipera* venom was found to contain 5-hydroxytryptamine (5-HT), commonly known as serotonin, which is well known as a nociceptive compound [164]. *G. marmoratus* venom was also found to contain 5-HT, or a 5-HT-like substance, that was found to act directly at 5-HT receptors and whose action was attenuated by a 5-HT receptor antagonist [76]. However, studies on stonefish venoms have failed to find evidence of serotonin [165,166].

Stonefish venoms have been found to contain norepinephrine, as well as substances that co-migrate with dopamine and tryptophan in electrochemical experiments. *S. verrucosa* venom was found to have relatively more norepinephrine when compared to *S. horrida*, but relatively less dopamine and tryptophan [165]. These compounds may contribute to the cardiovascular effects of stonefish venoms, although, as demonstrated by studies on SNTX and VTX, they are not the only, or even the major, factor.

A small non-proteinaceous toxin, with a molecular weight of 327 Da, was isolated from *P. volitans* venom and found to induce paralysis in killifish when in solution [30]. *P. volitans* venom was also found to contain acetylcholine, an although the role was unknown [74].

## 4. Conclusions

The evolution of venom in fish species has allowed them to adapt sedentary lifestyles and devolve squamation [56]. Instead of fleeing from predators they can simply erect their venomous spines. In order for the venom to act as an effective deterrent, it must be appropriately deleterious to potential predators. Thus fish have evolved highly potent toxins to act against vertebrate systems, as shown by the pharmacological studies examining the whole venoms and individual toxins of a variety of fish species.

Piscine venom studies have previously been largely focused on the broad pharmacology of whole venoms. Undoubtedly, a major factor in this was the technological limits at the time that the studies were conducted. While an important facet of understanding fish venoms, this means that the current knowledge on their composition is limited. Little is known about the number and ratio of individual components, as well as their structural characteristics and mechanisms of action. However, modern venomic methods have allowed for the creation of high-throughput pipelines to greatly increase the productivity of venom-based drug discovery [167]. These methods include high-throughput assays, transcriptomic analysis, and proteomic analysis involving highly sensitive mass spectrometry. Venomic approaches are currently being successfully used to study the venoms of conesnails [168], jellyfish [169], spiders [170], and snakes [171]. When combined with improved separation techniques for both large proteins and small peptides, applying these methods to fish venoms will modernize the field and create an effective approach to discovering and characterizing their components.

Despite the lack of comprehensive venom analysis using modern techniques, there has been some success in understanding piscine venoms. It appears that, as previously theorized [5], many fish venoms contain large toxins that may be causing a majority of the damage seen during envenomation. Toxins like Sp-CTx and SNTX form pores in cell membranes, causing depolarization and cell death. Cell components are then free to trigger the release of endogenous stores of neurotransmitters. The release of endogenous acetylcholine causes symptoms via action at muscarinic receptors and the release of endogenous catecholamines produce action at β-adrenoceptors, causing variations in blood pressure, respiration, and cardiac activity. Other components trigger the release of endogenous nitric oxide, which can then go on to increase vascular permeability and cause relaxation in smooth muscle. Additionally, the release of these endogenous stores results in neurotransmitter depletion at nerve terminals. This, along with depolarization of cell membranes due to the toxins can result in neuromuscular symptoms.

While responsible for several pharmacological activities, these toxins are unlikely to cause all of the symptoms of fish envenomation, especially those relating to nociception and inflammation. Indeed, stonefish toxin-like toxins may not even be present in many species, especially among non-scorpaeniformes. While these species may have toxins that work via a similar mechanism, the fact remains that fish venoms have been shown to contain a range of other toxins and enzymes. Although a number of these have been discovered, many more remain to be found and characterized. Additionally, many of the previously discovered toxins have yet to be fully characterized. As new methods and technologies develop and increase the rate at which biodiscovery may occur, it is important to continue investigation into piscine venoms using these methods to explore their potential as novel drug leads and physiological tools.
